# Association of Sleep Reactivity and Anxiety Sensitivity with Insomnia-Related Depression and Anxiety among City Government Employees in Japan

**DOI:** 10.3390/clockssleep5020015

**Published:** 2023-03-28

**Authors:** Isa Okajima, Hiroshi Kadotani, on behalf of the NinJa Sleep Study Group

**Affiliations:** 1 Behavioral Sleep Medicine and Sciences Laboratory, Department of Psychological Counseling, Faculty of Humanities, Tokyo Kasei University, Itabashi-ku, Tokyo 173-8602, Japan; 2 Department of Psychiatry, Shiga University of Medical Science, Seta Tsukinowa-cho, Otsu 520-2192, Japan; kadotanisleep@gmail.com

**Keywords:** sleep reactivity, anxiety sensitivity, insomnia, depression, anxiety

## Abstract

It has recently been noted that a reduction in sleep reactivity, characterized as the trait-like degree to which exposure to stress interferes with sleep, and anxiety sensitivity are associated with reduced insomnia severity. This study aimed to examine whether sleep reactivity and anxiety sensitivity are associated with insomnia-related depression and anxiety among city government employees in Japan. This cross-sectional study included 1810 city government employees of Koka City, Japan (mean age (standard deviation): 45.33 (12.20) years) who completely answered the scales for sleep reactivity, anxiety sensitivity, anxiety, and depression. Stepwise multiple regression analysis adjusted for demographic data showed that anxiety sensitivity (β = 0.39) was significantly linked to anxiety, and sleep reactivity (β = 0.36) was significantly linked to depression in individuals with insomnia. Additionally, the results of a logistic regression analysis adjusted for demographic data showed that anxiety sensitivity and sleep reactivity were relevant factors for anxious insomnia (OR = 12.69) and depressive insomnia (OR = 8.73), respectively. Whereas both sleep reactivity (OR = 14.67) and anxiety sensitivity (OR = 6.14) were associated with combined insomnia. These findings indicate that sleep reactivity is strongly associated with depressive symptoms, and anxiety sensitivity is strongly associated with anxiety symptoms in individuals with insomnia.

## 1. Introduction

Approximately 20–40% of the general population has been reported to experience insomnia symptoms [1,2,3], and nearly 17% of them experience chronic insomnia [3]. In addition, insomnia can present throughout one’s the lifespan and it can often be chronic [1,2], which is associated with the onset or recurrence of depression and resistance to depression treatment [4,5,6].

During the coronavirus disease 2019 (COVID-19) pandemic, insomnia became a serious issue. In an international collaborative study [7], 36.7% of the community sample complained of insomnia symptoms. Insomnia during the pandemic was associated with symptoms of posttraumatic stress and psychological distress [8]. In addition, infection anxiety during COVID-19 was associated with insomnia, depression, and anxiety [9]. Furthermore, individuals diagnosed with COVID-19 had a higher risk of a subsequent onset of insomnia disorder than those infected with other viruses (e.g., influenza) [10,11].

Cognitive behavioral therapy for insomnia (CBT-I) has been found to be an effective treatment and is recommended as a first-line intervention for chronic insomnia disorder [12,13,14]. A meta-analysis of the mediating factors on improving insomnia symptoms using CBT-I showed that changes in dysfunctional beliefs about sleep and pre-sleep arousal significantly mediated improvement in insomnia symptoms [15]. However, it has recently been noted that reduced sleep reactivity and anxiety sensitivity are associated with increased insomnia severity [16,17].

Sleep reactivity is characterized as the trait-like degree to which exposure to stress interferes with sleep, resulting in difficulty falling and staying asleep [18]. Individuals with a higher sleep reactivity showed a longer sleep latency, greater number of awakenings, and higher somatic arousal [19,20]. Recently, sleep reactivity has become an indicator of vulnerability to the onset of insomnia, with higher sleep reactivity related to an increased risk of depression [21], and insomnia functions as a mediator between sleep reactivity and depression [22]. It is also known that higher sleep reactivity is related to a higher severity of depression and anxiety during insomnia [23].

Anxiety sensitivity is defined as the fear of negative consequences associated with anxiety arousal, and it has been identified as a vulnerability to the onset of panic disorder [24,25]. It was one type of anxiety disorder characterized by repeated and frequently unanticipated panic attacks. Furthermore, anxiety sensitivity is considered a predisposing condition of insomnia [26], which is a mediating factor between sleep anticipatory anxiety and sleep latency [27], and decreases the frequency of sleep-related daytime impairment and using hypnotics [28].

Although there are no studies showing that sleep reactivity and anxiety sensitivity are associated with specific physical diseases, anxiety sensitivity has been found to be associated with pain severity in headaches [29].

It has been reported that these arousal factors are decreased by CBT-I. For example, digital CBT-I for workers with insomnia was more effective in reducing sleep reactivity (Hedges’ g = −1.09) compared to the wait-list control [30]. In addition, cognitive anxiety sensitivity treatment (CAST) targeted towards anxiety sensitivity is effective in improving insomnia via a reduction in anxiety sensitivity [17,31].

Sleep reactivity is likely to be associated with insomnia-related depression, and anxiety sensitivity is associated with insomnia-related anxiety. However, previous studies have examined depression and anxiety symptoms without adequately distinguishing between their symptoms. A systematic review revealed that insomnia predicted the onset of depression (odds ratio [OR] = 2.83) and anxiety (OR = 3.23) [6]. Furthermore, CBT-I is effective in treating insomnia with depression and anxiety comorbidities [32]. Therefore, it is assumed that a reduction in these arousal factors leads to an improvement, and subsequently, a reduction in the symptoms of symptoms depression and anxiety. If this hypothesis is proven, it becomes apparent which characteristics of anxiety sensitivity and sleep reactivity cause specific emotional problems. In particular, a tendency for an increased risk of mortality associated with the use of hypnotics in individuals with insomnia was reported [33]; the development and provision of more effective non-pharmacologic treatment is certainly important.

The present study aimed to examine whether sleep reactivity and anxiety sensitivity are associated with insomnia-related depression and anxiety among city government employees in Japan. We took advantage of this research to study the unique impact of sleep reactivity and anxiety sensitivity on specific emotions.

## 2. Results

**Figure 1:**
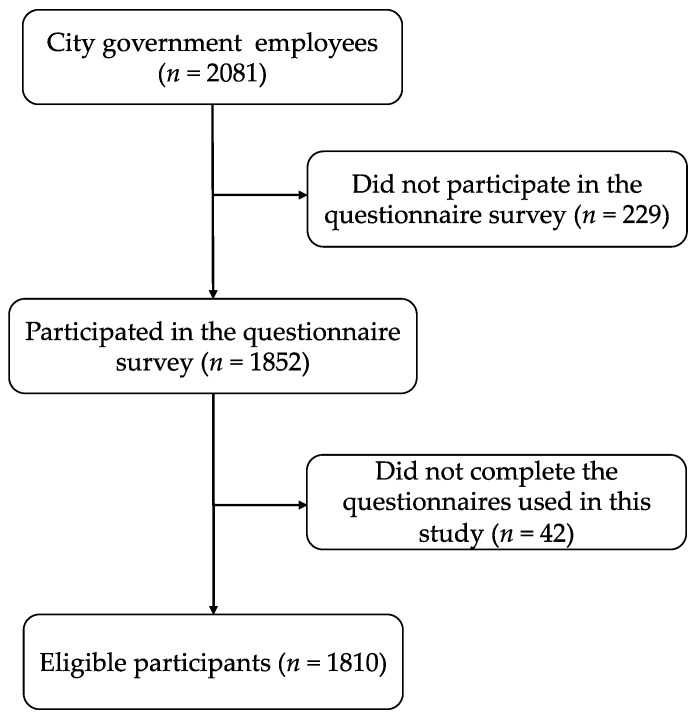
Study flowchart.

A total 1810 employees (699 males, 1111 females; mean age (standard deviation) 45.33 (12.20) years) completely responded to the measures (Figure 1). In this study, 4.4% were taking sleep medication, 3.3% antianxiety medication, and 2.4% antidepressants. Of them, 0.6% had cardiovascular infarction, 0.9% had cerebral infarction, and 11.7% were shift workers.

The participants were classified into five categories based on the cutoff scores for the Athens Insomnia Scale (AIS), Generalized Anxiety Disorder-7 (GAD-7), and Patient Health Questionnaire-9 (PHQ-9) are as follows: normal control, insomnia alone, anxious insomnia, depressive insomnia, and combined insomnia. The proportion of normal control was 52.5% and the insomnia category was 11.7%, which included insomnia alone, anxious insomnia, depressive insomnia, and combined insomnia. The proportions of patients with insomnia alone, anxious insomnia, depressive insomnia, and combined insomnia were 4.3%, 3.3%, 10.4%, and 82.0%, respectively. The descriptive statistics of all scales are presented in Table 1.

The exploratory factor analysis (EFA) on all the items of the Ford Insomnia Response to Stress Test (FIRST) and the Anxiety Sensitivity Index (ASI) revealed a two-factor structure; sleep reactivity (all items of the FIRST; factor loadings: 0.30–0.95) and anxiety sensitivity (all items of the ASI; factor loadings: 0.37–0.83). The correlation coefficient (r) between the factors was 0.46 (Table 2).

As the results of a stepwise multiple regression analysis adjusted for the demographic data in individuals with insomnia, for anxiety, that of ASI (β = 0.39, *p* < 0.001) was significantly linked to GAD-7 (R^2^ = 0.25 *p* < 0.001) in Step 1, and both ASI (β = 0.29, *p* < 0.001) and FIRST (β = 0.24, *p* < 0.001) were linked to GAD-7 in Step 2 (R^2^ = 0.30, *p* < 0.001), but the ΔR^2^ value was slightly increased (ΔR^2^ = 0.04; Table 3).

For depression, the FIRST (β = 0.36, *p* < 0.001) was related to PHQ-9 (R^2^ = 0.22, *p* < 0.001) in Step 1, and both the FIRST (β = 0.29, *p* < 0.001) and ASI (β = 0.16, *p* < 0.001) were significantly related to the PHQ-9 in Step 2 (R^2^ = 0.24, *p* < 0.001), but the ΔR^2^ value was slightly increased (ΔR^2^ = 0.02; Table 3).

The results of a logistic regression analysis adjusted for demographic data showed that ASI (OR = 12.69, *p* < 0.05) was a relevant factor for anxious insomnia, but FIRST (OR = 5.86, *p* = 0.052) was marginally significant. For depressive insomnia, FIRST (OR = 8.73, *p* < 0.001) was the only relevant factor, and both FIRST (OR = 14.67, *p* < 0.001) and ASI (OR = 6.14, *p* < 0.001) were related to combined insomnia (Table 4).

We conducted a post hoc power analysis using the results of a multiple regression analysis for GAD-7 and PHQ-9 in this study. With a regression *df*s of 10, the residual *df* of 198, the *f*^2^ [R^2^] of 0.43 [0.30] and 0.32 [0.24], and α = 0.001, the power (1 − β) were estimated at 0.99 and 0.99, respectively.

## 3. Discussion

This study aimed to examine whether sleep reactivity and anxiety sensitivity are associated with insomnia-related depression and anxiety among city government employees in Japan. The results showed that sleep reactivity was strongly associated with depressive symptoms such as hopeless, little energy, and poor appetite, and anxiety sensitivity was highly associated with anxiety symptoms such as nervousness, worry, and irritability.

In this study, pathological insomnia was defined as an AIS ≥ 10 points, and the proportion was approximately 12%. The prevalence of insomnia with daytime impairment has been reported to be approximately 17% [3]; the proportion of the individuals classified as insomniacs in this study is similar to that of the clinical level of insomnia.

The results of the EFA showed that sleep reactivity determined by FIRST and anxiety sensitivity identified by ASI were different constructs. This is remarkable because both sleep reactivity and anxiety sensitivity are regarded as predisposing factors for physiological arousal from insomnia.

The results of multiple regression analysis revealed that both sleep reactivity and anxiety sensitivity were associated with depression and anxiety symptoms in individuals with clinical level of insomnia. Considering the small ΔR^2^ value, sleep reactivity is particularly associated with depression, and anxiety sensitivity is related to anxiety. These findings are in line with those of previous studies [22,27]. Sleep reactivity directly and indirectly affects the onset of depression via the onset of insomnia [22], and anxiety sensitivity is a mediating factor between sleep anticipatory anxiety and sleep latency [27]. Similarly, the results of logistic regression analysis revealed that sleep reactivity was related to insomnia-related depression, while anxiety sensitivity was only associated with insomnia-related anxiety.

It is suggested that high levels of sleep reactivity and anxiety sensitivity might increase negative emotions in individuals with insomnia. Sleep reactivity is involved in a wide range of emotional responses in insomnia since sleep reactivity was also marginally associated with anxious insomnia. It is remarkable for predicting and preventing the subsequent onset of depression and anxiety disorders due to insomnia. CBT-I is an effective treatment for reducing insomnia severity and the mental symptoms of insomniacs with comorbid depression and anxiety disorders [34]. For example, the remission rates of CBT-I with eight weekly sessions were reported to be 57% at post-treatment and 56% at the 6-month follow-up [35]. In addition, reductions in sleep reactivity and anxiety sensitivity by CBT-I are likely to be significant mediators that reduce the severity of insomnia [16,17]. Thus, reducing both mediators as predisposing factors may prevent the recurrence of insomnia and improve insomnia-derived anxiety and depressive disorders.

Currently, a CAST with a single session has been developed as an approach to directly reduce anxiety sensitivity and improve insomnia symptoms by reducing anxiety sensitivity [31]; however, no direct approach for sleep reactivity has been developed. It is suggested that low/high sleep reactivity may be involved in the treatment response to CBT-I [16,18], although the reason for the reduction in sleep reactivity by CBT-I is not clear. In the future, an approach for the direct reduction in sleep reactivity should be developed and the mechanism of action should be examined.

This present study has some limitations. First, although this was a cross-sectional study, we were unable to identify causal relationships among sleep reactivity, anxiety sensitivity, insomnia, depression, and anxiety. Future prospective and longitudinal studies are needed to evaluate the influence of sleep reactivity and anxiety sensitivity on changes in insomnia-related depression and anxiety. Second, the participants were classified into insomnia categories using the cut-off scores for AIS, GAD-7, and PHQ-9, but not using the diagnostic criteria for these disorders. Therefore, additional studies are needed for patients with insomnia who have comorbid anxiety and/or depressive disorders. Third, participants in this study were limited to city government employees. It is necessary to confirm whether similar results can be obtained in a community sample in the future. Finally, we did not measure objective sleep parameters, such as electroencephalography. Depressive symptoms are associated with prolonged objective sleep and reduced rapid eye movement latency [36]. It is necessary to examine the relationship between sleep reactivity, anxiety sensitivity and the sleep architecture of depressive and anxious insomnia in the future.

## 4. Materials and Methods

### 4.1. Participants

A total of 2081 participants were government employees at Koka City, which is a rural city in Shiga Prefecture, Japan. The employees for whom the consent of a legal representative was required for participation were excluded from this study. Questionnaires were distributed to these employees, and a total of 1852 participants returned the questionnaire. Of these, 1810 employees completely responded to the measures (Figure 1). The questionnaire survey was conducted from 1 October 2021 to 18 March 2022.

### 4.2. Measures

#### 4.2.1. Demographic Data

We collected age, sex, a kind medication taken (hypnotics, antianxiety medication, and antidepressants), the presence of cardiovascular and neurological diseases, and shift-worker status as demographic data.

##### Insomnia-Related Measures

The Japanese version of the AIS was used to measure insomnia severity [37,38,39]. The scale consists of eight items, and the total score was calculated, with lower scores indicating a lower insomnia severity. The severity level of insomnia has been categorized as subthreshold (0–5 points), mild (6–9 points), moderate (10–15 points), and severe (≥16 points) [39]. When individuals received the AIS scores of ≥10 points, they were expected to be diagnosed with insomnia in the cohort study [5]; therefore, individuals with AIS scores of ≥10 points were considered to have psychopathological insomnia in the study.

The Japanese version of the FIRST was used to measure sleep reactivity [19,40]. The scale consists of 9 items, and the total score was calculated, with lower scores indicating less sleep reactivity to stress.

The Japanese version of the ASI was used to measure anxiety sensitivity [24,41]. The scale consists of 16 items, and the total score was calculated, with lower scores indicating less anxiety sensitivity.

#### 4.2.2. Anxiety and Depression

The Japanese versions of the GAD-7 [42,43] and PHQ-9 [44,45] were used to measure symptoms of anxiety and depression, respectively. The GAD-7 consists of seven items, the PHQ-9 consists of nine items, and both scales have been recommended to assess symptoms of anxiety and depression, as listed in the DSM-5 [46]. The total scores for both scales were calculated, with higher scores indicating more anxiety or depression. Cutoff scores of 5 were determined for both scales [42,44]; therefore, individuals with the GAD-7 or PHQ-9 scores of ≥5 points were considered to have psychopathological anxiety or depression in this study.

### 4.3. Procedure

A cross-sectional study was performed as part of the Night in Japan Home Sleep Monitoring (NinJaSleep) Study, which is an epidemiological study on sleep and mental health [36]. The study was approved by the ethics committee of Shiga University of Medical Science (R2017-111), and written informed consent was obtained from each participant.

### 4.4. Sample Size

The sample size was calculated based on a power analysis using an effect size f^2^, which was calculated with multiple regression analysis. With f^2^ = 0.15 (moderate effect size), α = 0.01, power (1 − β) estimated at 0.80, it was calculated that the total sample size was 160. Since, in the general population, it has been reported that nearly 17% of them experience chronic insomnia [3], we recruited a thousand participants or more.

### 4.5. Statistical Analysis

An exploratory factor analysis (EFA) using the maximum likelihood method with promax rotation was conducted to ensure that the FIRST and ASI are different constructs because the FIRST was associated with trait anxiety [40]. Then, to examine whether sleep reactivity and anxiety sensitivity are associated with anxiety or depressive symptoms in individuals with insomnia, we performed a stepwise multiple regression analysis adjusted for demographic data with the FIRST and ASI as independent variables and the GAD-7 or PHQ-9 as dependent variables. An effect size for R^2^ value of >0.02 indicates a small effect, >0.13 indicates a moderate effect, and >0.26 indicates a large effect [47]. In addition, the ΔR^2^ value of >0.07 indicates a medium effect size [48].

The participants were classified into five categories based on the cutoff scores for AIS, GAD-7, and PHQ-9, which are as follows: normal control (AIS ≤ 9, GAD-7 ≤ 4, and PHQ-9 ≤ 4), insomnia alone (AIS ≥ 10, GAD-7 ≤ 4, and PHQ-9 ≤ 4), anxious insomnia (AIS ≥ 10, GAD-7 ≥ 5, and PHQ-9 ≤ 4), depressive insomnia (AIS ≥ 10, GAD-7 ≤ 4, and PHQ-9 ≥ 5), and combined insomnia (AIS ≥ 10, GAD-7 ≥ 5, and PHQ-9 ≥ 5). In addition, sleep reactivity and anxiety sensitivity were divided into high and low groups based on the mean scores for the FIRST and ASI, respectively. Using these classifications, we performed a logistic regression analysis with normal control versus anxious insomnia, normal control versus depressive insomnia, and normal control versus combined insomnia as dependent variables, and high/low sleep reactivity and anxiety sensitivity as independent variables, adjusted for demographic data.

The data were analyzed using SPSS Version 26.0 (IBM Inc., Tokyo, Japan).

## 5. Conclusions

Sleep reactivity was strongly related to depressive symptoms, and anxiety sensitivity was highly associated with anxiety symptoms in city government employees with insomnia in Japan. The findings have important implications for preventing the onset and recurrence of anxiety disorders and depression with insomnia.

## Figures and Tables

**Table 1:** Descriptive statistics (means and SD) for all scales.

Scales	Total	Normal	Insomnia Alone	Anxious Insomnia	Depressive Insomnia	Combined Insomnia
Sleep reactivity (FIRST)	20.55 (6.45)	17.90 (5.78)	20.89 (4.91)	22.00 (7.07)	22.68 (7.15)	26.87 (4.83)
Anxiety sensitivity (ASI)	18.12 (12.13)	13.83 (10.74)	25.33 (12.48)	20.86 (10.96)	18.68 (8.89)	29.73 (12.85)
Insomnia (AIS)	5.14 (3.73)	2.99 (2.16)	10.56 (0.53)	11.00 (1.41)	11.36 (1.71)	12.81 (2.55)
Anxiety (GAD-7)	3.84 (4.20)	1.15 (1.24)	1.44 (1.33)	5.57 (1.13)	2.55 (1.22)	10.98 (4.20)
Depression (PHQ-9)	4.87 (4.73)	1.57 (1.36)	2.67 (1.58)	3.86 (0.38)	8.68 (3.17)	13.37 (4.64)

AIS, Athens Insomnia Scale; ASI, Anxiety Sensitivity Index; FIRST, Ford Insomnia Response to Stress Test; GAD-7 Generalized Anxiety Scale-7; PHQ-9, Patient Health Questionnaire-9; SD, standard deviation.

**Table 2:** Results of exploratory factor analysis.

	Factor Loadings	
Items	Factor 1	Factor 2
Factor 1: Anxiety sensitivity		
ASI 10	0.83	−0.05
ASI 16	0.81	0.00
ASI 12	0.80	−0.01
ASI 14	0.77	0.04
ASI 15	0.77	0.02
ASI 9	0.77	−0.06
ASI 6	0.76	0.06
ASI 8	0.74	0.04
ASI 4	0.72	0.04
ASI 3	0.71	0.00
ASI 11	0.71	−0.03
ASI 13	0.67	−0.09
ASI 2	0.53	0.05
ASI 5	0.42	0.15
ASI 7	0.41	0.15
ASI 1	0.37	0.10
Factor 2: Sleep reactivity		
FIRST 3	−0.07	0.95
FIRST 2	−0.04	0.93
FIRST 4	−0.01	0.86
FIRST 6	−0.01	0.86
FIRST 7	0.02	0.78
FIRST 1	0.03	0.70
FIRST 8	0.09	0.61
FIRST 5	0.12	0.43
FIRST 9	0.15	0.30
Factor correlation		
Factor 1	—	0.46

ASI, Anxiety Sensitivity Index; FIRST, Ford Insomnia Response to Stress Test.

**Table 3:** Multiple regression analysis of factors associated with anxiety and depression in individuals with insomnia.

	Anxiety (GAD-7)	Depression (PHQ-9)
**Step 1**		
Sleep reactivity (FIRST)	–	0.36 **
Anxiety sensitivity (ASI)	0.39 **	–
R^2^	0.25	0.22
**Step 2**		
Sleep reactivity (FIRST)	0.24 **	0.29 **
Anxiety sensitivity (ASI)	0.29 **	0.16 **
R^2^ (ΔR^2^)	0.30 (0.04)	0.24 (0.02)

ASI, Anxiety Sensitivity Index; FIRST, Ford Insomnia Response to Stress Test; GAD-7, Generalized Anxiety Scale-7; PHQ-9, Patient Health Questionnaire-9. Adjusted for demographic data. ** *p* < 0.001.

**Table 4:** Logistic regression analyses of factors associated with insomnia subtypes.

Variables	Anxious Insomnia OR [95% CI]	Depressive Insomnia OR [95% CI]	Combined Insomnia OR [95% CI]
Sleep reactivity (FIRST)	5.86 [0.97, 35.33]	8.73 ** [2.68, 28.40]	14.67 ** [8.30, 25.93]
Anxiety sensitivity (ASI)	12.69 * [1.34, 120.48]	1.83 [0.71, 4.75]	6.14 ** [3.79, 9.95]

ASI, Anxiety Sensitivity Index; FIRST, Ford Insomnia Response to Stress Test; OR, odds ratio; CI, confidence interval. Adjusted for demographic data. * *p* < 0.05, ** *p* < 0.001.

## Data Availability

The data presented in this study are available on request from the corresponding author.
